# Improving final diagnosis in paediatric posterior fossa tumours: Correlation and discrepancy between radiologic diagnosis, intraoperative surgeon’s diagnosis, and histopathology

**DOI:** 10.12669/pjms.41.13(PINS-NNOS).13363

**Published:** 2025-12

**Authors:** Muhammad Nawaz khan, Arshad Khan, Tahir Mehmood, Adnan Khan, Muhammad Sohaib Khan

**Affiliations:** 1 Muhammad Nawaz Khan, (MBBS, FCPS, DHPE).Assistant Professor & HoD, Department of Neurosurgery, Lady Reading Hospital-MTI, Peshawar, KPK, Pakistan; 2 Arshad Khan (MBBS, FCPS, MPH, M.Phil).Registrar, Department of Neurosurgery, Lady Reading Hospital-MTI, Peshawar, KPK, Pakistan; 3 Adnan Khan (MBBS, FCPS-1).Resident Neurosurgery, Department of Neurosurgery, Lady Reading Hospital-MTI, Peshawar, KPK, Pakistan; 4 Muhammad Sohaib Khan, (MBBS, FCPS-1).Resident Neurosurgery, Department of Neurosurgery, Lady Reading Hospital-MTI, Peshawar, KPK, Pakistan; 5 Tahir Mehmood, (PhD Biostatistics).Assistant Professor, School of Natural Sciences,National University of Sciences & Technology (NUST), Islamabad, Pakistan

**Keywords:** Diagnostic Discrepancy, Diagnostic Correlation, Radiologic Diagnosis, Posterior Fossa Tumours, Histopathologic Diagnosis, Intraoperative Surgeon Diagnosis

## Abstract

**Objectives:**

This study investigates discrepancies between histopathologic, intraoperative, and radiologic diagnosis of paediatric posterior fossa tumours and evaluates whether combining diagnostic modalities improves the accuracy of the final diagnosis.

**Methodology:**

This retrospective study was conducted at the Department of Neurosurgery, Lady Reading Hospital, Peshawar. Hospital Management Information System (HMIS) records of all paediatric patients, aged birth to 16 years, who underwent surgery in the department for paediatric posterior fossa tumours between January 2020 and December 2024, were reviewed. Radiologic diagnosis, surgeon intraoperative diagnosis and combined diagnosis (surgeon diagnosis + radiologic diagnosis) were compared with the final histopathologic diagnosis to calculate discrepancy rates.

**Results:**

A total of 112 paediatric patients were enrolled in the study. The discrepancy rate for radiologic diagnosis, surgeon (intraoperative) diagnosis and combined diagnosis (radiologic diagnosis + surgeon diagnosis) remained 19.64%, 21.43% and 7.14% respectively. The concordance analysis using Cohen’s Kappa statistic concluded that radiologic diagnosis and surgeon (intraoperative) diagnosis both showed moderate agreement with histopathology having Cohen’s kappa (k) values of 0.738 and 0.714 respectively while combined diagnosis exhibited the highest concordance (κ = 0.905) with histopathology.

**Conclusion:**

Combining radiologic and intraoperative surgeon diagnoses enhances accuracy in paediatric posterior fossa tumours, achieving the highest concordance with histopathology and reducing discrepancies. This combined approach represents best practice to improve diagnostic precision, especially in LMICs where multidisciplinary tumour boards are not readily available.

## INTRODUCTION

Discrepancies between radiologic diagnoses (MRI brain reported), intraoperative surgeon diagnoses and histopathologic diagnoses present significant challenges in clinical practice while dealing with brain tumours in general and paediatric posterior fossa tumours in particular. MRI reports for brain tumours often contain interpretative errors, potentially impacting surgical planning and outcomes. Diagnostic errors in neuroimaging reports for brain tumours range between 15% and 30%, primarily due to interpretative errors.^1^,^2^ Furthermore, histopathologic diagnosis occasionally is inconsistent with the surgeon’s intraoperative findings, necessitating a review by pathologists. Histopathological errors in central nervous system tumour diagnoses occur in approximately 5% to 15% of cases, directly influencing clinical management.^3^,^4^

These discrepancies highlight the diagnostic dilemma faced by neurosurgeons. Despite the collaborative nature of neuro-oncology, the final responsibility lies with the operating surgeon, who must integrate multiple sources of information—including surgeon interpretation of radiological images, formal radiology reports, intraoperative findings, and histopathological reports to provide a conclusive diagnosis to the patient for guidance on further treatment and prognosis. In the majority of low- and middle-income countries (LMICs), including Pakistan, formal tumour boards are not available due to lack of neuro-oncology and radiation oncology services in the same hospital where neurosurgical patients are operated.^5^ Hence, pre-operative and post-operative discussion on each and every brain tumour case in neuro-oncology multidisciplinary team (MDT) meeting is practically not feasible. Surgeons often request radiologists or histopathologists to review and revise their reports when inconsistencies arise, but ultimately it remains the surgeon’s responsibility to synthesize the data and communicate the final diagnosis. This underscores the critical need for enhanced diagnostic precision, continuous communication among specialties, and the establishment of diagnostic protocols & algorithms to mitigate diagnostic errors and improve patient care.

This study aimed to contribute to the existing body of knowledge by providing valuable insights into the diagnostic challenges. It explores the diagnostic discrepancies among histopathologic, surgeon’s intraoperative, and radiologic diagnoses in paediatric posterior fossa tumours. The study also proposes a diagnostic approach aimed at minimizing these discrepancies to achieve a more accurate final diagnosis.

## METHODOLOGY

This Retrospective study was conducted at the Department of Neurosurgery, Lady Reading Hospital, Peshawar. Hospital Management Information System (HMIS) records of all paediatric patients, aged birth to 16 years—who underwent surgery in the department for paediatric posterior fossa tumours between January 2020 and December 2024, were reviewed. Non probability sampling (purposive sampling) technique was used for patient selection. A total of 112 paediatric patients were enrolled in the study. All radiology reporting, histopathology evaluations and intraoperative surgeon diagnoses were conducted by faculty at the level of assistant professor or higher.

### Ethical approval:

It was given by the Institutional Review Board of Lady Reading Hospital, Peshawar through letter No. 107/LRH Hospital dated February 25, 2025.

### Inclusion criteria:

Patients who had undergone surgery for posterior fossa tumour and had preoperative 1.5 Tesla MRI brain with contrast and Diffusion Weighted Images (DWI) as well as radiologist report, surgical diagnosis (intraoperative findings), and postoperative histopathology report available in HMIS were included in the study.

### Exclusion criteria:

Patients with incomplete diagnostic data (missing surgeon intraoperative findings, MRI report or histopathology report), recurrent posterior fossa tumours, or those previously treated with radiotherapy/chemotherapy were excluded from study.

### Data analysis technique:

Intraoperative diagnosis recorded by surgeons in operating notes— describing characteristics such as consistency, vascularity, margins, and adherence to surrounding structures or a brief surgical diagnosis was considered acceptable for inclusion in the study. Post-operative histopathology reports without documented suspicion of discrepancy by operating surgeon were considered as valid and final diagnosis. While for those histopathology reports with documented discrepancy suspicion by operating surgeon, review histopathology reports were considered as final diagnosis. Data collection included demographics and diagnostic data. ***Statistical analysis:*** Statistical analysis included descriptive statistics, concordance analysis using Cohen’s Kappa statistic, diagnostic accuracy, subgroup analysis, and discrepancy analysis.

## RESULTS

The study involved a cohort of 112 paediatric patients (66 males and 46 females) who underwent surgery for posterior fossa tumours, with ages ranging from birth to 16 years (mean 9.3 ± 3.2 years). A significant portion of the patients (72%, n=81) were under 12 years of age. Gender-wise, statistical analysis showed no significant difference in tumour distribution (p = 0.32). However, subgroup analysis showed male predominance in medulloblastoma (p = 0.02), while pilocytic astrocytoma showed no gender bias (p = 0.84).

### Histopathologic findings:

The initial histopathology diagnoses of all 112 cases revealed medulloblastoma as the most common tumour (60 cases), followed by pilocytic astrocytoma (20 cases), ependymoma (12 cases), and AT/RT and brainstem glioma (10 cases each).

The overall discrepancy analysis of initial versus review histopathology reports for paediatric posterior fossa tumours revealed an average discrepancy rate of 27.67%. The highest discrepancy was observed in AT/RT cases (60%), while medulloblastoma showed the lowest discrepancy (11.67%).

### Radiology diagnostic performance:

**Fig.1:**
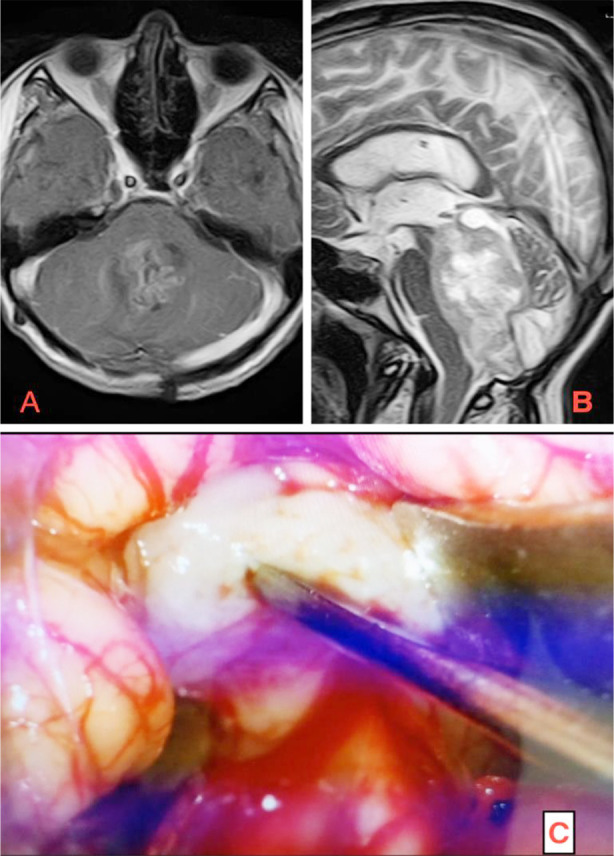
Paediatric posterior fossa tumor radiologically reported as medulloblastoma, with intraoperative features suggestive of ependymoma, and histopathology confirming ependymoma. A: axial T1-weighted contrast enhanced MRI image, B: T2-weighted sagittal MRI, C: intraoperative appearance of a pale white, soft tumour.

Radiologic (MRI brain reported) diagnoses were compared to the histopathologic diagnoses. As shown in Table-I, MRI misdiagnosed six cases of medulloblastoma (discrepancy 10%), followed by three cases of pilocytic astrocytoma (discrepancy 15%), four cases each of ependymoma (discrepancy 33.33%) and AT/RT (discrepancy 40%), and 05 cases of brainstem glioma (discrepancy 50%). Diagnostic accuracy for MRI brain showed high sensitivity for medulloblastoma (90%), while sensitivity for other tumour types ranged from 50% to 85%. A total of 19.64% discrepancy rate in the initial radiologic (MRI reported) diagnoses suggests a need for improved diagnostic accuracy through second-opinion and re-evaluation. An example of one such patient is demonstrated in Fig.1.

**Table-I:** Diagnostic performance of radiologic (MRI reported) diagnoses relative to histopathologic diagnoses (final diagnoses).

Histopathologic Diagnoses		Radiologic Diagnoses			
Tumour types	No of Cases (n)	Correct MRI Diagnoses	Incorrect MRI Diagnoses	Discrepancy (%)	Sensitivity (%)
Medulloblastoma	60	54	6	10%	90%
Pilocytic Astrocytoma	20	17	3	15%	85%
Ependymoma	12	8	4	33.33%	66.67%
AT/RT	10	6	4	40%	60%
Brainstem Glioma	10	5	5	50%	50%
Total	112	90	22	19.64%	80.36%

### Surgeon (Intraoperative) Diagnostic Performance:

As stated in Table-II, the surgeon (intraoperative) diagnoses compared to the valid histopathological diagnoses (after obtaining second opinion if surgeon documented discrepancy/suspicion) demonstrated high sensitivity for medulloblastoma (85%), pilocytic astrocytoma (75%) and ependymoma (75%); though sensitivity for brainstem glioma and AT/RT was lower at 60% and 70% respectively. The overall accuracy of intraoperative diagnosis was moderate (Discrepancy rate 21.43), reflecting the difficulties in making definitive intraoperative diagnosis.

**Table-II:** Diagnostic Performance of Surgeon Diagnoses Relative to Histopathologic Diagnoses.

Histopathologic Diagnoses		Surgeon Diagnoses			
Tumour types	No of Cases (n)	Correct Surgeon Diagnoses	Incorrect Surgeon Diagnoses	Discrepancy (%)	Sensitivity (%)
Medulloblastoma	60	51	9	15%	85%
Pilocytic Astrocytoma	20	15	5	25%	75%
Ependymoma	12	9	3	25%	75%
AT/RT	10	7	3	30%	70%
Brainstem Glioma	10	6	4	40%	60%
Total	112	88	24	21.43%	78.57%

### Combined Diagnostic Performance:

As depicted by the statistics in the Table-III, if we combine radiologic (MRI reported) diagnoses and surgeon (intraoperative) diagnoses, the accuracy and sensitivity become comparable to histopathologic diagnosis, while the discrepancy percentage significantly decreases for each type of tumour.

**Table-III:** Combined Diagnostic Performance relative to Histopathologic Diagnoses

Histopathologic Diagnoses		Combined Diagnosis			
Tumour type	No of Cases (n)	Correct combined Diagnosis	Incorrect combined Diagnosis	Discrepancy (%)	Sensitivity (%)
Medulloblastoma	60	57	3	5%	95%
Pilocytic Astrocytoma	20	19	1	5%	95%
Ependymoma	12	11	1	8.33%	91.67%
AT/RT	10	8	2	20%	80%
Brainstem Glioma	10	9	1	10%	90%
Total	112	104	8	7.14%	90.33%

### Statistical analysis and concordance:

**Fig.2:**
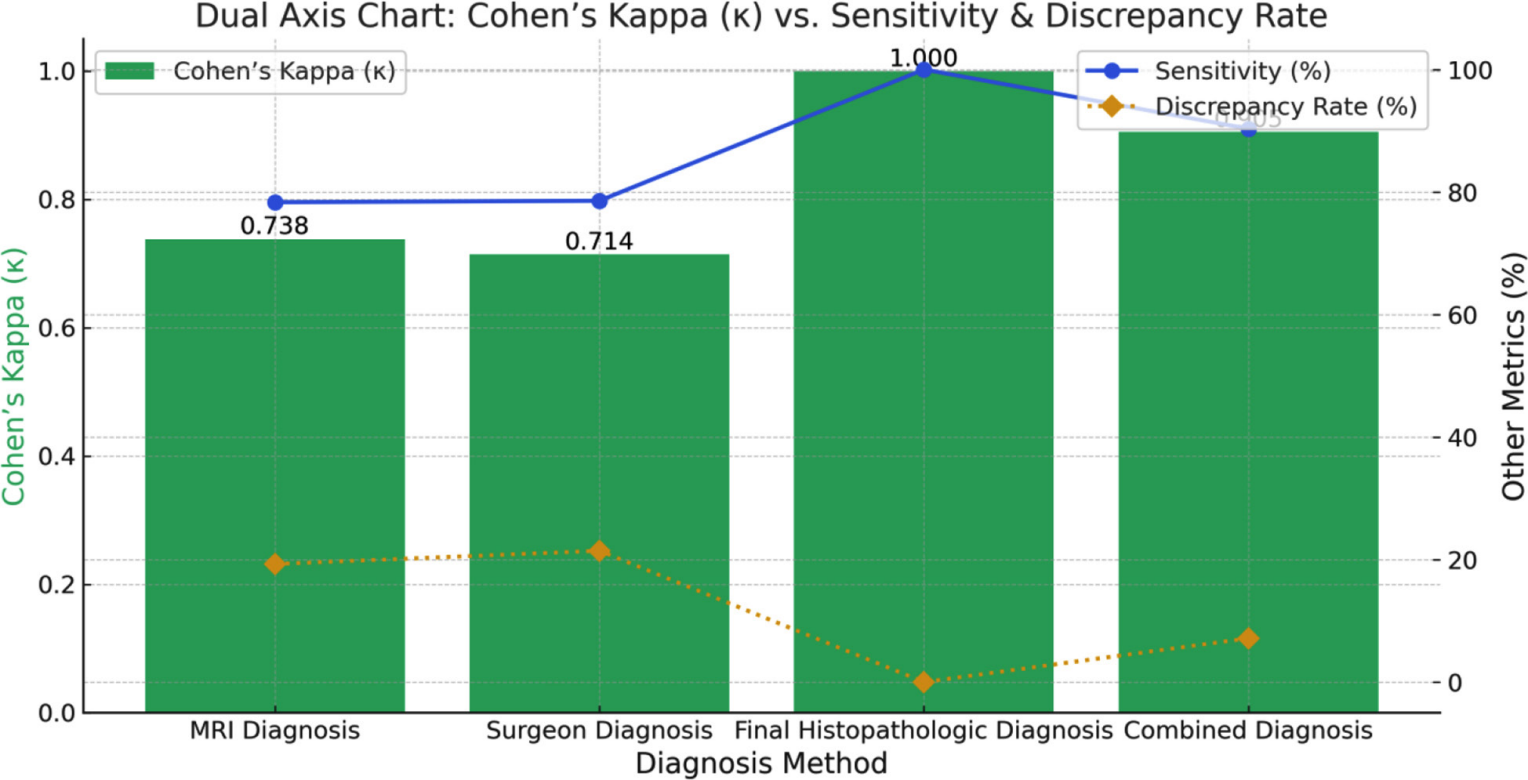
Dual-axis chart, where Cohen’s Kappa (κ) is shown as a bar chart. Sensitivity and Discrepancy Rate are displayed as line graphs for comparison.

Radiologic (MRI reported) diagnoses and surgeon (intraoperative) diagnoses both showed moderate agreement with histopathology having Cohen’s kappa (k) values of 0.738 and 0.714 respectively while combined diagnoses (Radiologic plus surgeon diagnoses) exhibited the highest concordance with histopathology (κ = 0.905), with the lowest discrepancy rate (7.14%). This suggests that a combined approach of diagnosis can improve diagnostic accuracy, Fig.2.

### Subgroup analysis:

Subgroup analysis revealed that tumours larger than 5cm had a higher diagnostic accuracy (85%) compared to smaller tumours (75%). Additionally, younger patients (<10 years) had lower diagnostic accuracy (82%) compared to older patients (≥10 years), who had an accuracy of 88%. Soft, cystic tumours exhibited higher accuracy in diagnosis compared to solid, vascular tumours.

## DISCUSSION

The statistical analysis further confirmed the overall diagnostic discrepancy across all the modalities. The sensitivity of radiologic diagnosis ranged from 50% to 90%. In comparison, surgeon (intraoperative) diagnosis sensitivity ranged from 60% to 85%. The sensitivity values combining both modalities were reasonably high, approaching 95%, highlighting that radiologic plus surgeon diagnosis is good at accurately identifying the tumour type. Similar studies by Chiaravalloti A et al. and Wu HW et al. reported that, although MRI is highly sensitive for detecting brain tumours, its specificity in diagnosing specific tumour types—particularly in the posterior fossa—remains limited.^6^,^7^

Radiologic diagnosis independently showed a moderate level of concordance with histopathology (κ = 0.738), but also revealed a discrepancy rate of 19.64%. This finding is in agreement with previous research by Resende LL et al. and Kapadia T et al., who highlighted the high sensitivity of MRI in detecting brain tumours. However, it faces limitations in terms of specificity when distinguishing between different posterior fossa tumours.^8^,^9^ Jaju A et al. observed that medulloblastomas and AT/RTs often show overlapping radiological features on conventional MRI brain images, making it challenging to differentiate them solely based on imaging results.^10^ The discrepancy rate of 19.64% observed in our study highlights this challenge.

The intraoperative diagnosis made by surgeons also showed moderate agreement with histopathology, with a κ value of 0.714 and a discrepancy rate of 21.43%. This moderate level of concordance is reflective of the inherent difficulties in intraoperative diagnosis, particularly when dealing with diffuse and infiltrative tumours such as brainstem gliomas. In the case of well-circumscribed tumours and consistent morphology like medulloblastomas, the accuracy of intraoperative diagnosis is typically higher. These findings are consistent with the work of Uribe-Cardenas R et al., who mentioned that three factors play key role in the precision of intraoperative diagnosis, i.e. tumour location, surgeon’s experience and the tumours` typical presentation.^11^

The combined approach of radiologic and intraoperative diagnosis led to a substantial reduction in the discrepancy rate, bringing it down to just 7.14%, with a Cohen’s Kappa (κ) value of 0.905—indicating near-perfect agreement, the highest among all diagnostic methods. These results are consistent with studies by Foster MT et al. and Colafati et al., who stated that combining MRI diagnosis and intraoperative neurosurgical observations increases diagnostic concordance in complex paediatric brain tumour cases.^12^-^14^ The combined approach enables surgeons to integrate radiology reports with intraoperative tumour characteristics assessment to decide about initial histopathology report discrepancies. If suspicion arises, the surgeon may ask the pathologist for a second opinion on the tissue block, which is considered the final diagnosis.

The subgroup analysis provided further insights into the factors affecting diagnostic accuracy. Larger tumours (>5 cm) in older children (>10 years age) diagnosed with 85% accuracy, compared to 75% for smaller tumours in younger patients. This discrepancy may stem from the difficulty of interpreting imaging in younger patients, whose smaller brain structures and atypical tumour presentations can complicate accurate diagnosis, as postulated by Young RJ et al. in their study.^15^,^16^

Overall, a combined radiologic and intraoperative surgeon findings approach enhances diagnostic accuracy more than either modality alone.

### Limitations:

The small sample size and single-center retrospective design, relying on recorded clinical data, and potential interobserver variability may limit the generalizability of our findings.

## CONCLUSION

Combining preoperative radiologic diagnosis with the surgeon’s intraoperative observations significantly improves diagnostic accuracy for paediatric posterior fossa tumours, achieving near-perfect concordance with histopathology and reducing discrepancies. Where feasible, this integrated diagnostic approach should be considered best practice. In LMICs, protocols can help compensate for the limited availability of tumour boards.

### Clinical recommendations:

Further studies involving multiple centers and large sample sizes of diverse populations would provide better results to be adopted and applied to improve the accuracy of diagnosing paediatric posterior fossa tumours.

### Authors’ Contributions:

**MNK:** Concept and design and critical review.

**AK:** Data Collection and manuscript drafting. Accountable for the of the study.

**TM:** Data Analysis and critical review.

**AK MSK:** Data acquisition and manuscript writing.

All authors have read and approved the final version of the manuscript.
